# The mediating role of altruistic leadership perception in the relationship between psychological wellbeing and job performance among coaches

**DOI:** 10.3389/fpsyg.2026.1781192

**Published:** 2026-03-27

**Authors:** Ramazan Arslanboga, Muhammed Ozkan Turhan, Sultan Yavuz Eroglu, Mustafa Can Koc, Laurentiu-Gabriel Talaghir, Gabriel Marian Manolache, Teodora Mihaela Iconomescu, Florentina Cristea

**Affiliations:** 1 Faculty of Sports Sciences, Bingöl University, Bingöl, Türkiye; 2 Faculty of Sports Sciences, Mus Alparslan University, Mus, Türkiye; 3 Faculty of Sports Sciences, Istanbul Gelisim University, Istanbul, Türkiye; 4 Faculty of Physical Education and Sport, Dunarea de Jos University of Galati, Galati, Romania

**Keywords:** altruistic leadership, coaches, job performance, mediating role, wellbeing

## Abstract

**Background:**

The mediating role of altruistic leadership perception in the relationship between psychological wellbeing and job performance has not yet been sufficiently investigated in samples of coaches.

**Objective:**

This study aimed to examine the mediating role of altruistic leadership perception in the relationship between psychological wellbeing and job performance among coaches.

**Methods:**

The research sample consisted of 491 coaches actively working in the Eastern Anatolia region who participated voluntarily and was selected using a simple random sampling method. The participants were between 18 and 44 years old, with a mean age of 26.6 ± 7.37 years. As data collection instruments, the Short Form of the Psychological WellBeing Scale developed by Telef, the Altruistic Leadership Scale originally developed by Barbuto and Wheeler and adapted into Turkish by Çakmak et al., and the Job Performance Scale developed by Çalişkan and Köroglu were administered. Data were analyzed using SPSS and Jamovi software. The scales used in the study were validated, and their construct validity was confirmed.

**Results:**

The findings revealed statistically significant positive associations among psychological wellbeing, altruistic leadership, and job performance, with the strongest association observed between psychological wellbeing and job performance.

**Conclusions:**

Mediation analysis indicated that coaches' psychological wellbeing showed a strong direct association with job performance, while altruistic leadership demonstrated a small but statistically significant indirect effect within this relationship. From a theoretical perspective, this study contributes to the sports management and organizational psychology literature by empirically demonstrating that altruistic leadership may function as a meaningful relational pathway linking psychological wellbeing and job performance in coaching contexts.

## 1 Introduction

Today, the sustainability of organizations depends not only on the availability of material resources but also on the effective protection and management of human resources. One of the prominent approaches within this framework is altruistic leadership. Altruistic leaders adopt an empathy-based leadership style that prioritizes the development and wellbeing of employees without seeking personal gain (Kaya and Ekinci, 2025). With these characteristics, altruistic leadership is regarded as an effective approach due to its contributions to various types of motivation and work processes. When examining the manifestations of leadership in different contexts, sports organizations stand out as an important field of application. In particular, the performance of coaches in sports directly reflects both individual and organizational success. Considering that sports encompass not only physical but also psychological and social dimensions, the leadership styles and behavioral patterns of coaches play a decisive role in athletes' motivation, team cohesion, and sustainable success (Arman, 2025). Likewise, studies in various sectors reveal that leadership styles significantly affect employee performance (Aga et al., 2016; Aktuna and Kiliçlar, 2019; Eraslan and Demir, 2020). In this context, the capacity of leadership approaches to guide coaches' professional development processes emerges as a noteworthy element.

Psychological wellbeing is a multidimensional concept that encompasses elements such as individuals' satisfaction with life, the attribution of meaning to life, the experience of positive emotions, and the ability to cope with encountered challenges (Dooris et al., 2021). Individuals with high levels of psychological wellbeing demonstrate stronger commitment to their work, engage more effectively in decision-making processes, and significantly enhance their performance (Rego and Pina e Cunha, 2008; Wright and Cropanzano, 2004). The literature frequently emphasizes that leadership practices support employees' psychological wellbeing, which in turn positively influences job performance (Chaudhary et al., 2022; Maheshwari et al., 2024; Zeike et al., 2019). Within this framework, the impact of psychological wellbeing on job performance applies not only to organizational structures but also to professionals working in sports.

Coaches represent a unique professional group within sports organizations, as they simultaneously perform leadership, mentoring, and performance-oriented roles. Their dual responsibility for both relational and performance outcomes makes them particularly suitable for examining the interplay between psychological wellbeing, leadership behaviors, and job performance. Therefore, investigating these mechanisms in coaching contexts provides a theoretically meaningful extension of organizational research into sport-specific environments. Coaches, in particular, represent key actors in whom this relationship is embodied, aligning with the existing literature (Amelda et al., 2021; Artüz and Bayraktar, 2021; Mohamed, 2022). The literature also emphasizes that psychological wellbeing plays a decisive role in domains of critical importance to organizations, such as creativity, innovation, commitment, achievement, and job performance (Garcea et al., 2010). From a theoretical perspective, this study is grounded in positive organizational behavior, which emphasizes the role of individual psychological resources in shaping work-related outcomes (Luthans, 2002; Wright and Cropanzano, 2004). Psychological wellbeing can be conceptualized as a personal resource that enhances individuals' cognitive, emotional, and behavioral capacities in professional settings. Within this framework, leadership behaviors may function as relational mechanisms through which individual psychological resources are translated into performance outcomes. Despite the growing body of research on leadership, psychological wellbeing, and job performance, these constructs have largely been examined independently or through direct associations. Limited attention has been given to understanding the process-based mechanisms through which psychological wellbeing may translate into performance outcomes in sports organizations. In particular, the potential mediating role of altruistic leadership in coaching contexts remains underexplored. Addressing this gap, the present study integrates these variables within a unified mediational framework to provide a more comprehensive understanding of performance dynamics in sports management settings. The importance of this study lies in its integrative examination of psychological wellbeing, altruistic leadership, and job performance within coaching contexts, thereby extending leadership and organizational psychology research into sport-specific environments and offering a more process-oriented understanding of performance determinants in sports management. Based on the findings of previous studies, the first hypothesis of this study was formulated as follows:

**H**_**1**_**:** Coaches' psychological wellbeing is positively associated with their job performance.

According to existing studies, individuals with high levels of psychological wellbeing tend to feel more confident and comfortable with their leaders' decisions and behaviors (Özdemir and Yirmibeş, 2016). However, when reviewing the literature, no research has been found that integrates the concepts of psychological wellbeing and altruistic leadership within the coaching context. Nevertheless, in other disciplines, several studies have identified a relationship between wellbeing and leadership (Erschens et al., 2022; Kim and Cruz, 2022; Özkan et al., 2022; Gyu Park et al., 2017). Based on the available evidence, it can be inferred that coaches with higher levels of psychological wellbeing perform their profession not merely as a duty but in a more meaningful and value-oriented manner. This perspective is considered to form the foundation of altruistic leadership. On the other hand, the theoretical basis of the model proposed in this study aims to explain how the relationship between psychological wellbeing and job performance is shaped through leadership perception. Resource Conservation Theory emphasizes that individuals tend to conserve and develop their psychological and social resources. According to this theory, when faced with stress, employees develop responses aimed at preventing resource loss. Individuals experience stress when they perceive a threat to their available resources, when resources are actually lost, or when expended resources cannot be adequately compensated (Orhan, 2022). In this context, coaches with high levels of psychological wellbeing can be expected to exhibit more altruistic leadership behaviors. The hypotheses of the study, developed within the framework of theory and conceptualization, are as follows.

**H**_**2**_**:** Coaches' psychological wellbeing is positively associated with their altruistic leadership.

One of the key determinants of organizational success is job performance. Job performance refers to the degree to which tasks are performed effectively and efficiently, and it is regarded as a critical factor influencing both individual and organizational outcomes. Indeed, high levels of employee performance contribute to enhancing organizational capacity and competitiveness, while also supporting sustainability (Aktuna and Kiliçlar, 2019). Leadership, defined by various researchers as the process of directing individuals toward specific goals, motivating them, and facilitating their success (Aktuna and Kiliçlar, 2019; Black and Porter, 2000; Krill and Carter, 1997; Ribière and Sitar, 2003), also plays a central role in this regard. Social change theory suggests that employees who have a high-quality leader-member exchange relationship tend to be more effective workers (Walumbwa et al., 2011). This process suggests that leadership perception can enhance job performance through mutual interaction and trust. On the other hand, individuals are expected to reflect the positive behaviors they observe in others positively toward other individuals. Consequently, the notion that leadership styles affect employee performance is frequently supported in the literature. Drawing on these insights, it can be inferred that coaches who display high levels of altruistic leadership are likely to enhance their work performance, as trust and commitment constitute the core of their relationships with both their organizations and athletes. Therefore, the third hypothesis was formulated as follows:

**H**_**3**_**:** Coaches' altruistic leadership is positively associated with their job performance.

Coaches play critical roles in multidimensional processes ranging from athletes' individual development to the organizational success of sports clubs (Güllü, 2018; Yildiz, 2009). Hence, a comprehensive understanding of the factors influencing coaches' job performance is strategically important not only for athletes but also for the overall success of sports organizations (Akbiyikli and Sürgevil, 2020; Yildiz et al., 2019). Considering the impact of leadership and psychological factors on job performance, it is evident that coaches' leadership styles and psychological wellbeing levels play a decisive role in athletes' motivation and team success. However, ongoing discussions regarding whether this influence occurs directly or through specific psychological mechanisms have gained increasing attention. Thus, it is becoming essential to examine the underlying structure of the leadership-performance relationship comprehensively. In this context, investigating psychological wellbeing as a mediating variable in the relationship between altruistic leadership perception and job performance offers both theoretical contributions to the literature and practical implications for improving coaches' professional processes (Ateş and Ateş, 2023; Güler and ve Yildiz, 2023). The Positive Organizational Behavior approach supports the idea that wellbeing can lead to increased performance outcomes by nurturing positive perceptions of leadership. It involves the examination and application of measurable, developable, and efficiently manageable positive traits and psychological capacities for performance development in today's organizations (Luthans et al., 2006). Therefore, it can be explained within a theoretical framework that psychological wellbeing guides leadership behaviors and functions as an intermediary mechanism that strengthens the relationship between leadership and wellbeing performance. While psychological wellbeing may directly associated with performance, it is thought that this effect can be strengthened through altruistic leadership behaviors. Accordingly, the fourth hypothesis was proposed as follows:

**H**_**4**_**:** Altruistic leadership is expected to play a mediating role in the association between coaches' psychological wellbeing and their job performance.

The main objective of this study is to determine the effect of coaches' psychological wellbeing on their job performance and to examine whether altruistic leadership behaviors play a mediating role in this relationship. Furthermore, it aims to explain the reflections of coaches' leadership behaviors on both performance and wellbeing. For this reason, the theories addressed in the H1, H2, and H3 hypotheses explain the resource management dimension of psychological wellbeing through the Conservation of Resources Theory, while the Social Change Theory reveals how this wellbeing is reflected in performance through leader-member interactions. Positive Organizational Behavior demonstrates that this process can be supported by measurable and developable psychological capacities at the organizational level.

## 2 Materials and methods

### 2.1 Research model

**Figure 1:**
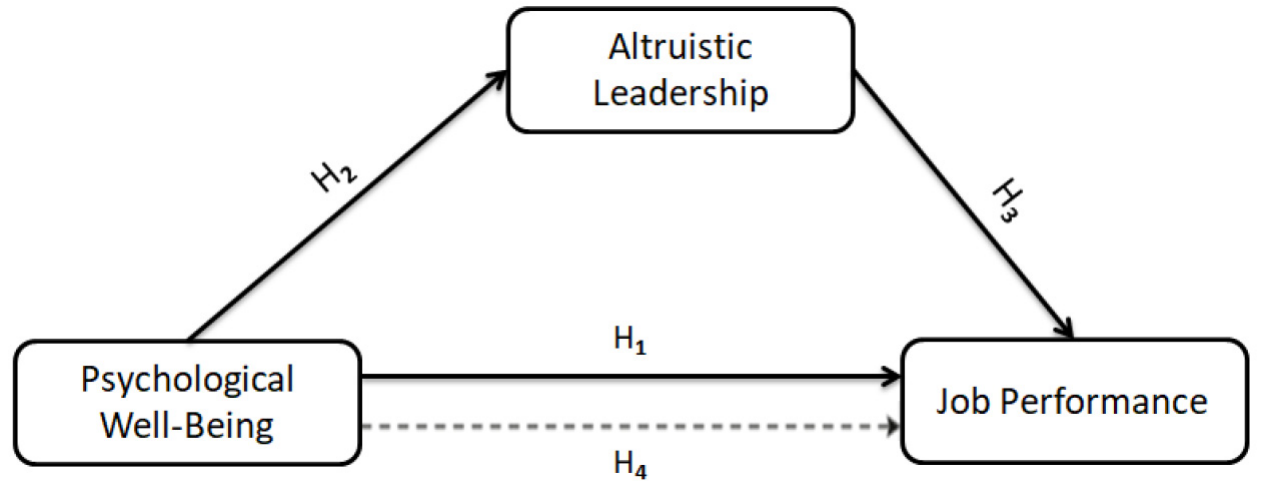
Research model.

This study employed a cross-sectional correlational research design. The correlational survey model is used to examine the associations among variables and to determine the extent to which they vary together (Karasar, 2018). In line with the theoretical framework of the study (see Figure 1), the associations among coaches' psychological wellbeing, altruistic leadership, and job performance were analyzed within a mediation model.

### 2.2 Population and sample

To determine the minimum sample size for the study, an a priori power analysis was conducted using G^*^Power 3.1 software. The analysis family selected was the “linear multiple regression: fixed model, R^2^ increase” test, consistent with the regression-based mediation model. The analysis was based on a medium effect size (*f*^2^ = 0.150), a 95% confidence level (α = 0.050), and a 95% test power (1–β = 0.950). The minimum required sample size was calculated as 107 (Cohen, 2013). The target population consisted of coaches actively working in sports organizations within the Eastern Anatolia Region. Participants were selected using a simple random sampling approach. Data were collected through both face-to-face administration and electronic distribution to increase accessibility and participation. Participation was entirely voluntary. The research group consisted of 491 coaches actively working in the Eastern Anatolia Region, selected through simple random sampling, who voluntarily participated in the study. The participants' ages ranged from 18 to 44, with a mean age of 26.6 ± 7.37. Regarding professional experience, 77.4% of the participants had 0–5 years of coaching experience, 14.5% had 6–10 years, 4.9% had 11–15 years, and 3.3% had 16 or more years of experience. This distribution indicates that the sample predominantly consisted of early-career coaches. Simple random sampling is one of the most fundamental probability sampling methods, in which each individual in the population has an equal chance of being selected (Büyüköztürk et al., 2017; Karasar, 2018). Although efforts were made to enhance representativeness through probability sampling and multiple data collection methods, the findings should be interpreted within the regional context in which the data were collected. The participants' personal information is presented in Table 1.

**Table 1:** Information regarding participants' individual characteristics.

Variables	Groups	Frequency (n)	Percentage (%)	X
Gender	Male	348	70.9	
	Female	143	29.1	
Age				26.6
Marital status	Married	102	20.8	
	Single	389	79.2	
Coaching experience	0–5 years	380	77,4	
	6–10 years	71	14,5	
	11–15 years	24	4,9	
	16+ years	16	3,3	

### 2.3 Data collection tools

Data were collected using the personal information form prepared by the researchers, the Psychological WellBeing Scale (PWS-8), the Altruistic Leadership Scale, and the Job Performance Scale.

#### 2.3.1 Personal information form

Data regarding participants' demographic characteristics, including gender, age, and marital status, were collected using a personal information form prepared by the researchers.

#### 2.3.2 Psychological wellbeing scale (PWS-8)

The Psychological WellBeing Scale Short Form (PWS-8), developed by Telef (2013), was used to assess participants' levels of psychological wellbeing. The scale consists of eight items, has a unidimensional structure, and is rated on a 5-point Likert-type scale (1 = Strongly Disagree, 5 = Strongly Agree). Higher scores indicate higher levels of psychological wellbeing. In validity and reliability analyses, the Cronbach's alpha coefficient was found to be 0.86. Confirmatory factor analysis results demonstrated that the scale's unidimensional structure provided an acceptable fit (χ^2^*/df* = 2.10, RMSEA = 0.056, CFI = 0.97, GFI = 0.96, AGFI = 0.94, SRMR = 0.041).

#### 2.3.3 Altruistic leadership scale

To measure participants' perceptions of leadership, the Altruistic Leadership Scale, originally developed by Barbuto and Wheeler (2006) and adapted into Turkish by Çakmak et al. (2019), was employed. The scale consists of eight items and has a unidimensional structure. Participants rated the statements using a 5-point Likert-type scale (1 = Never, 5 = Always). Confirmatory factor analysis indicated that the model demonstrated a good fit with the data (NFI = 0.975, TLI = 0.976, IFI = 0.987) (Table 2). The Cronbach's alpha coefficient was calculated as 0.927, and item-total correlations ranged between 0.59 and 0.82.

**Table 2:** Fit index values for the data collection tools.

Data collection too	*χ2/df*	RMSEA	CFI	TLI	SRMR
Altruistic leadership scale	3.84	0.076	0.96	0.95	0.02
	(Acceptable)	(Acceptable)	(Good fit)	(Good fit)	(Good fit)
Psychological wellbeing scale	3.88	0.077	0.96	0.95	0.03
	(Acceptable)	(Acceptable)	(Good fit)	(Good fit)	(Good fit)
Job performance scale	4.07	0.079	0.95	0.94	0.03
	(Acceptable)	(Acceptable)	(Good fit)	(Good fit)	(Good fit)
Acceptable fit^*^	≤ 5	< 0.08	>0.90	>0.90	< 0.08
Good fit^*^	≤ 3	< 0.05	>0.97	>0.95	< 0.05

^*^Indicates emphasis and level of significance.

#### 2.3.4 Job performance scale

The Job Performance Scale developed by Çalişkan and Köroglu (2022) was used to assess participants' job performance perceptions. The scale consists of 11 items grouped under two subscales (Task Performance and Contextual Performance) and is rated on a 5-point Likert-type scale (1 = Strongly Disagree, 5 = Strongly Agree). There are no reverse-coded items in the scale. Confirmatory factor analysis results indicated acceptable fit indices (χ^2^*/df* = 1.72, RMSEA = 0.06, NFI = 0.93, GFI = 0.91, AGFI = 0.88, CFI = 0.97). In reliability analyses, the Cronbach's alpha coefficient was found to be 0.871.

### 2.4 Analysis of data

Data collected from the coaches were analyzed using the SPSS software package. Prior to analysis, z-scores were computed for each item to detect outliers. Eighteen participants with z-scores greater than 3.30 were excluded from the dataset. After validating the scale structures, skewness and kurtosis values were examined for normality, and these values were found to range between –2 and +2 (Tabachnick and Fidell, 2007). Pearson correlation analysis was conducted to examine the relationships among variables, and the coefficients were interpreted as follows: 0.00–0.10 insignificant, 0.10–0.39 weak, 0.40−0.69 moderate, 0.70−0.89 strong, and 0.90–1.00 very strong (Schober et al., 2018). In the next stage, mediation analyses were conducted using the JAMOVI 2.5.4 software. The significance of mediation effects was evaluated using the bootstrap method (Gürbüz and Bayik, 2021). Accordingly, for a 95% confidence interval, the effect is considered significant if the lower and upper bounds do not include zero and if the *p-value* is less than 0.05 (Arbuckle, 2011).

## 3 Result

Of the participants, 348 (70.9%) were male and 143 (29.1%) were female. The mean age of the participants was 26.6 years. In terms of marital status, 102 participants (20.8%) were married and 389 (79.2%) were single.

Confirmatory factor analysis (CFA) was conducted to test the construct validity of the measurement tools. Based on the modification indices provided by the statistical software, a limited number of covariance terms between items belonging to the same latent construct were added. These modifications were implemented only when conceptually and theoretically justifiable. In line with Simşek's (2007) perspective that correlating error terms within the same latent variable does not compromise constructs validity, the adjustments were retained. As a result, the model demonstrated acceptable and good fit values (Meydan and Seşen, 2015; Schumacker and Lomax, 2015).

As part of the reliability analysis of the scales, the AVE (Average Variance Extracted) and CR (Composite Reliability) values were calculated for each factor. The online AVE and CR calculation system developed by Aydogdu (2023) was used for these computations. An AVE value greater than 0.50 was considered the acceptable threshold for convergent validity, while a CR value greater than 0.70 was accepted as the threshold for composite reliability (Fornell and Larcker, 1981). The skewness and kurtosis values of the variables ranged between –2 and +2, confirming the assumption of normal distribution (Tabachnick and Fidell, 2007). The Cronbach's alpha coefficient ranges from 0 to 1, and a value of at least 0.70 is considered acceptable for reliability (Field, 2009; Hair et al., 2019). Furthermore, McDonald's omega (ω) is another reliability coefficient calculated in addition to Cronbach's alpha; as it approaches 1, it indicates that the obtained scores are more reliable (Lucke, 2005). Pearson correlation analysis was conducted to examine the relationships among the variables. The results indicated moderate positive and significant associations between psychological wellbeing and job performance (*r* = 0.49, *p* < 0.05), and between psychological wellbeing and altruistic leadership (*r* = 0.43, *p* < 0.05). Additionally, a strong positive and significant association was observed between altruistic leadership and job performance (*r* = 0.70, *p* < 0.05) (Table 3).

**Table 3:** Interrelationships between variables, reliability coefficients (α, ω, CR), average variance extracted (AVE), and skewness-kurtosis values.

S. No	Variables	*(α)*	*(ω)*	CR	AVE	Skewness/Kurtosis	M	SD	1	2
1	Altruistic leadership	0.88	0.88	0.91	0.57	−0.047/−0.092	3.67	0.80	–	
2	Psychological wellbeing	0.88	0.89	0.89	0.50	−0.078/0.200	5.65	0.98	0.49^*^	–
3	Job performance	0.92	0.92	0.92	0.52	−0.050/−0.175	4.23	0.58	0.43^*^	0.70^*^

^*^Indicates emphasis and level of significance.

The direct associations of psychological wellbeing on job performance and the indirect associations of psychological wellbeing on job performance through altruistic leadership were both significant. Moreover, 7.88% of the total associations of psychological wellbeing on job performance was indirect, whereas 92.12% was direct. In the mediation effect analysis performed with the bootstrap method, for the research hypothesis to be supported, the values in the 95% confidence interval (CI) must not include zero (MacKinnon et al., 2004). The findings indeed showed that the confidence interval values did not include zero (see Figure 1). In this context, coaches' psychological wellbeing levels are associated with their job performance, and altruistic leadership appears to mediate this relationship (Table 4).

**Table 4:** Mediation effect analysis.

Effect	Label	Estimate	SE	95% confidence interval		*Z*	*p*	% Mediation
				Lower	Upper			
Indirect	a × b	0.044	0.001	0.015	0.078	2.79	< 0.005	7.88
Direct	c	0.523	0.003	0.451	0.590	14.63	< 0.001	92.12
Total	c + a × b	0.568	0.003	0.505	0.629	17.80	< 0.001	100

Psychological wellbeing was positively associated with altruistic leadership (β = 0.403; *p* < 0.001). Altruistic leadership was positively associated with job performance [β = 0.111; *p* < 0.005; 95% CI (0.037, 0.191)]. Furthermore, psychological wellbeing showed a positive association with job performance both directly [β = 0.523; *p* < 0.001; 95% CI (0.451, 0.591)] and indirectly through altruistic leadership. Although the indirect effect was statistically significant (Table 5), its magnitude was relatively small compared to the direct effect, suggesting that altruistic leadership plays a limited but meaningful mediating role.

**Table 5:** Effect coefficients.

Predictor		Outcome	Estimate	SE	95% confidence interval		*Z*	*p*
					Lower	Upper		
Psychological wellbeing	➡	Altruistic leadership	0.403	0.033	0.337	0.471	12.08	< 0.001
Altruistic leadership	➡	Work performance	0.111	0.039	0.037	0.191	2.83	< 0.005
Psychological wellbeing	➡	Job performance	0.523	0.035	0.451	0.591	14.63	< 0.001

The conceptual model of the study is presented in Figure 1.

**H**_**1**_**:** Coaches' psychological wellbeing is positively associated with their job performance.
**H**_**2**_**:** Coaches' psychological wellbeing is positively associated with their altruistic leadership.
**H**_**3**_**:** Coaches' altruistic leadership is positively associated with their job performance.
**H**_**4**_**:** Altruistic leadership is expected to play a mediating role in the association between coaches' psychological wellbeing and their job performance.

**Figure 2:**
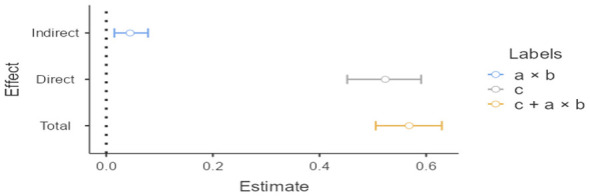
Confidence intervals.

The findings presented in Figure 2 show that the confidence interval values did not include zero.

**Figure 3:**
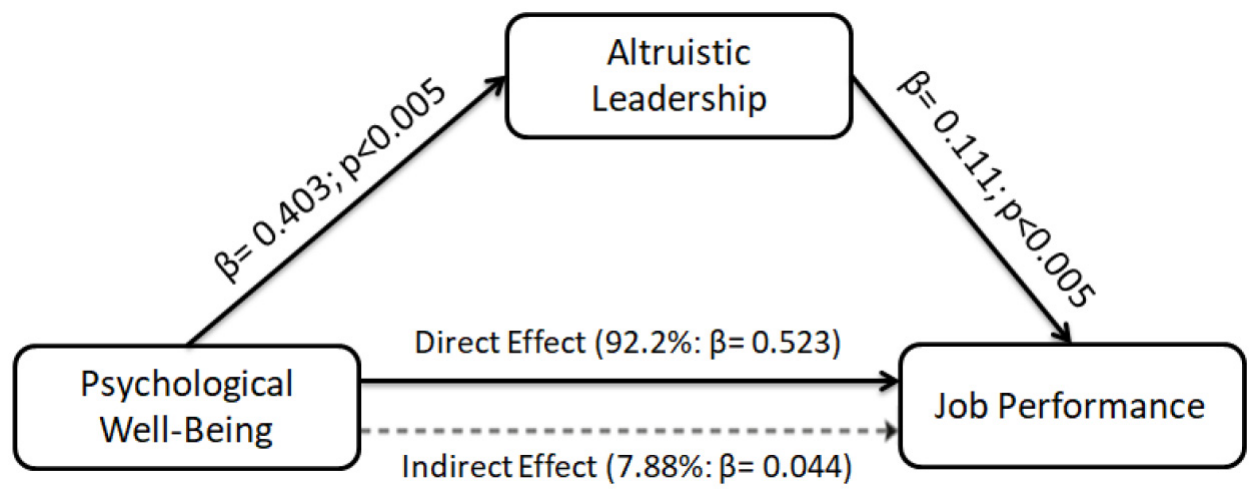
Mediated effect model.

As illustrated in Figure 3, the findings indicate that, coaches' psychological wellbeing was strongly and positively associated with on their job performance. In addition, altruistic leadership showed a low-level positive indirect association with job performance.

## 4 Discussion

This study aimed to examine the mediating role of altruistic leadership behaviors in the relationship between coaches' psychological wellbeing levels and job performance, in accordance with the information presented in the literature. The analyses revealed that coaches' psychological wellbeing was positively associated with job performance, both directly and indirectly through altruistic leadership. While the direct association was relatively strong, the indirect association through altruistic leadership represented a smaller proportion of the total effect. This finding indicates that psychological wellbeing is an effective factor in enhancing job performance on its own, yet it also offers an additional mechanism of influence through altruistic leadership. The findings obtained are consistent with both positive psychology-based approaches and mediation models discussed in the leadership literature. In Wright and Cropanzano's (2000) study, psychological wellbeing was defined as a key variable predicting job performance. Furthermore, studies examining the associations of positive leadership approaches on employee wellbeing similarly emphasize the importance of considering the interaction between leadership and employee wellbeing (Azila-Gbettor et al., 2024). These findings demonstrate that organizational employees' adoption of leadership approaches supports psychological wellbeing and can play an important role in enhancing job performance. The altruistic leadership style examined in the study can therefore be considered an important factor that indirectly strengthens the associations of psychological wellbeing on performance.

Below, the findings related to each hypothesis are discussed in light of the literature.

**H**_**1**_**:** Coaches' psychological wellbeing is positively associated with their job performance.

The data obtained within the scope of the research revealed that psychological wellbeing had a direct and strong associations on job performance (β = 0.523; *p* < 0.001). This finding supports the relevant hypothesis (H_1_). Wright and Wright and Cropanzano's (2000) study also indicated that psychological wellbeing, along with job satisfaction, positively predicts job performance. Similarly, the study titled Psychological WellBeing and Job Satisfaction as Predictors of Job Performance concluded that psychological wellbeing directly increases job performance and that job satisfaction partially mediates this effect (Yang et al., 2024). These results are consistent with the current findings. When evaluated in the context of sports, high levels of psychological wellbeing can positively influence coaches' job performance by supporting their decision-making, stress management, and relationship-building skills under physical and psychosocial pressures. From a broader organizational and managerial perspective, it has also been revealed that, in addition to technical competence, measures should be implemented to support coaches' psychological wellbeing by reducing risk factors such as burnout and fatigue. This is considered essential for ensuring the sustainability of coaching performance and for further advancing the field of sports. From an effect size perspective, the magnitude of this association (β = 0.523) can be considered substantial in behavioral research contexts, where effect sizes in organizational and sport settings are often moderate due to the multicausal nature of performance outcomes (Funder and Ozer, 2019). This finding therefore reflects not only statistical significance but also meaningful practical relevance within coaching environments.

**H**_**2**_**:** Coaches' psychological wellbeing is positively associated with their altruistic leadership.

The analyses showed that psychological wellbeing had a moderately positive associations on altruistic leadership (β = 0.403; *p* < 0.001). This finding aligns with the literature suggesting that individuals with high psychological wellbeing are more likely to demonstrate empathetic and supportive behaviors that prioritize the wellbeing of others. Indeed, studies examining the relationship between leadership behaviors and employees' psychological wellbeing emphasize that leadership approaches can enhance employees' levels of wellbeing (Inceoglu et al., 2018). Similarly, Messias et al. (2010) also reported that leadership behaviors have a significant impact on employees' psychological wellbeing. The findings in the literature are consistent with our study, and hypothesis H_2_ is therefore supported. When evaluated in the context of sports, it can be stated that coaches with high levels of psychological wellbeing can strengthen team cohesion by displaying helpful, empathetic, and supportive leadership behaviors toward athletes.

**H**_**3**_**:** Coaches' altruistic leadership is positively associated with their job performance.

The analysis results (β = 0.111; *p* < 0.005) showed that altruistic leadership had a positive but relatively low. The analyses showed that psychological wellbeing had a moderately positive associations on altruistic leadership on job performance, and hypothesis H_3_ was supported. This finding suggests that the relationship between leadership and performance is shaped by more complex mechanisms rather than a direct and strong. The analyses showed that psychological wellbeing had a moderately positive associations on altruistic leadership, and that effective leadership styles can increase performance when supported by other psychological processes. The study titled The Effects of Leadership Behaviors on Employee WellBeing in the Workplace emphasized that leadership behaviors have significant effects on employees' attitudes, performance, and psychological wellbeing, yet mediating models play a decisive role in this process (Inceoglu et al., 2018). Similarly, Kelloway et al. (2013) indicated that positive leadership behaviors enhance employees' psychological wellbeing and that this improvement is reflected in performance-related outcomes. In this context, although altruistic leadership alone may not generate high levels of performance, it can strengthen its influence when interacting with psychological wellbeing. In the sports context, coaches who exhibit altruistic leadership can foster elements such as trust, commitment, and morale; however, the impact of these contributions on performance becomes more pronounced when supported by individuals' levels of psychological wellbeing. Although the standardized coefficient (β = 0.111) indicates a relatively small direct association, small effect sizes are common in complex social and performance-based contexts where multiple variables simultaneously influence outcomes (Lakens, 2013). In sport psychology, leadership effects on performance are often indirect, cumulative, and context-dependent rather than immediately large in magnitude. Therefore, even modest coefficients may reflect meaningful relational contributions that unfold over time within team dynamics and coaching environments (Inceoglu et al., 2018). In sport psychology research, associations between leadership behaviors and performance outcomes are often modest in magnitude, reflecting the multifactorial and situational nature of athletic performance (Arthur et al., 2017; Vella et al., 2013). Therefore, the relatively small coefficient observed in the present study should be interpreted within this broader performance context, where leadership typically operates through relational and motivational processes rather than as a sole determinant of performance.

**H**_**4**_**:** Altruistic leadership is expected to play a mediating role in the association between coaches' psychological wellbeing and their job performance.

The analyses revealed that 7.88% of the total associations of psychological wellbeing on job performance was mediated by altruistic leadership, while 92.12% occurred directly. This finding indicates that altruistic leadership plays a limited but meaningful mediating role. In other words, while psychological wellbeing directly and strongly affects job performance, altruistic leadership contributes to explaining a certain portion of this relationship. This result aligns with other studies in the literature employing mediation models. For instance, Inceoglu et al. (2018) examined the effects of leadership behaviors on employees' psychological wellbeing, emphasizing the crucial role of mediation models in this process. Similarly, Azila-Gbettor et al. (2024) stated that positive leadership approaches enhance employees' wellbeing levels and that this improvement is reflected in job performance. This literature support reinforces the validity of the finding obtained under H_4_. Although indirect effects accounted for only 7.88% of the total effect, when the importance of this effect is evaluated from the perspective of sports psychology, it is thought that selfless leadership is a sensitive and meaningful way that leads to increased work performance when coaches are psychologically well. This situation suggests that small indirect effects can accumulate over time and influence interactions within the team, athletes' motivation, and the organizational environment. This finding sheds light on the process described in social interaction theory, which suggests that leadership perception can enhance work performance through mutual interaction and trust relationships. Therefore, the mediating role of selfless leadership is an important source of information showing how psychologically positive states produce practical results in coaching environments. Importantly, mediation effects in cross-sectional organizational research are frequently modest in size, particularly when the independent variable exerts a strong direct association, as observed in the present study. Contemporary methodological discussions emphasize that indirect effects should be evaluated in terms of process explanation rather than magnitude alone (MacKinnon et al., 2004; Funder and Ozer, 2019). In coaching contexts, relational leadership behaviors may operate as incremental mechanisms translating psychological resources into performance-related outcomes. Thus, while the indirect effect accounted for 7.88% of the total association, its explanatory contribution remains theoretically meaningful. Furthermore, in applied sport settings, small indirect effects may represent meaningful process-based mechanisms that accumulate over time within team dynamics and coaching interactions (Arthur et al., 2017). Thus, although the mediating effect accounted for a limited proportion of the total association, it contributes to explaining how psychological resources are translated into performance-related outcomes in relational sport environments.

## 5 Conclusions

In summary, this study highlights the mediating role of altruistic leadership perception in the association between psychological wellbeing and job performance among coaches.

At the policy level, sports federations and governing bodies may consider incorporating psychological wellbeing components into coach certification and licensing systems. Sports clubs and administrators may consider implementing structured initiatives aimed at enhancing coaches' psychological wellbeing, including stress management programs, mentoring systems, and accessible psychological support mechanisms. Embedding such initiatives within formal coach education curricula and continuous professional development programs may help institutionalize psychological resource development rather than treating it as an isolated intervention.

Leadership training frameworks may place greater emphasis on empathy, altruism, and supportive behaviors to strengthen relational dynamics within teams. Incorporating applied components-such as scenario-based training, feedback systems, and athlete-centered communication workshops-may facilitate the practical development of altruistic leadership behaviors.

Performance evaluation systems may also incorporate wellbeing and leadership-related indicators alongside technical skills and outcome-based criteria. Integrating relational and psychological dimensions into performance assessment processes may contribute to more sustainable and holistic performance management models within sports organizations.

Future research could compare different sports, cities, or countries and employ longitudinal designs to examine changes over time. Including additional mediating or moderating variables, such as self-efficacy, organizational commitment, or psychological capital, could provide a more comprehensive understanding of these relationships.

### 5.1 Limitations of the study

The sample for this study is limited to coaches in Turkey's Eastern Anatolia region. Given that this regional and cultural context may influence perceptions of leadership and psychological wellbeing, the generalizability of the findings to other cultural or organizational contexts is limited. Cultural values, norms, and expectations can shape how altruistic leadership is perceived and how psychological wellbeing is reflected in job performance. Therefore, conducting future research in different cultural contexts and with more diverse samples will increase the external validity of the findings. On the other hand, since all variables were collected simultaneously and through self-reporting, there is a risk of common method variance (CMV). However, no diagnostic test has been applied for this risk. Therefore, the findings should be interpreted with this limitation in mind.

Another limitation of the study concerns the use of self-report instruments for measuring all variables. Although validated scales were employed and confidentiality was ensured to reduce response bias, reliance on self-reported data may increase the risk of social desirability bias and common method variance. Future studies may benefit from incorporating multi-source assessments, such as athlete evaluations or supervisor ratings, as well as objective performance indicators.

Additionally, information regarding specific sport branches (e.g., team vs. individual sports) was not collected. Future studies may examine whether the proposed relationships differ across sport types or competitive contexts.

## Data Availability

The raw data supporting the conclusions of this article will be made available by the authors, without undue reservation.
